# Characterization of hepatic fatty acids using magnetic resonance spectroscopy for the assessment of treatment response to metformin in an eNOS^−/−^ mouse model of metabolic nonalcoholic fatty liver disease/nonalcoholic steatohepatitis

**DOI:** 10.1002/nbm.4932

**Published:** 2023-04-11

**Authors:** Begoña Lavin, Thomas R. Eykyn, Alkystis Phinikaridou, Aline Xavier, Shravan Kumar, Xabier Buqué, Patricia Aspichueta, Carlos Sing‐Long, Marco Arrese, René M. Botnar, Marcelo E. Andia

**Affiliations:** ^1^ School of Biomedical Engineering Imaging Sciences King's College London London UK; ^2^ BHF Centre of Research Excellence, Cardiovascular Division King's College London London UK; ^3^ Department of Biochemistry and Molecular Biology, School of Chemistry Complutense University Madrid Spain; ^4^ Biomedical Engineering, Faculty of Engineering Universidad de Santiago de Chile Santiago Chile; ^5^ ANID ‐ Millennium Science Initiative Program ‐ Millennium Institute Intelligent Healthcare Engineering Santiago Chile; ^6^ Physiology Department, School of Medicine and Nursing Universidad del País Vasco UPV/EHU Vizcaya Spain; ^7^ Biocruces Bizkaia Health Research Institute Barakaldo Spain; ^8^ CIBER de enfermedades hepáticas y digestivas (CIBERehd) Spain; ^9^ School of Engineering Pontificia Universidad Católica de Chile Santiago Chile; ^10^ Gastroenterology Department Faculty of Medicine, Pontificia Universidad Católica de Chile Santiago Chile; ^11^ School of Medicine and Centro de Envejecimiento y Regeneración (CARE), Facultad de Ciencias Biológicas Pontificia Universidad Católica de Chile Santiago Chile

**Keywords:** eNOS^−/−^, metformin, MRI, NAFLD, thin‐layer chromatography

## Abstract

Nonalcoholic fatty liver disease (NAFLD) is the leading cause of chronic liver disease worldwide. Liver biopsy remains the gold standard for diagnosis and staging of disease. There is a clinical need for noninvasive diagnostic tools for risk stratification, follow‐up, and monitoring treatment response that are currently lacking, as well as preclinical models that recapitulate the etiology of the human condition. We have characterized the progression of NAFLD in eNOS^−/−^ mice fed a high fat diet (HFD) using noninvasive Dixon‐based magnetic resonance imaging and single voxel STEAM spectroscopy‐based protocols to measure liver fat fraction at 3 T. After 8 weeks of diet intervention, eNOS^−/−^ mice exhibited significant accumulation of intra‐abdominal and liver fat compared with control mice. Liver fat fraction measured by ^1^H‐MRS in vivo showed a good correlation with the NAFLD activity score measured by histology. Treatment of HFD‐fed NOS3^−/−^ mice with metformin showed significantly reduced liver fat fraction and altered hepatic lipidomic profile compared with untreated mice. Our results show the potential of in vivo liver MRI and ^1^H‐MRS to noninvasively diagnose and stage the progression of NAFLD and to monitor treatment response in an eNOS^−/−^ murine model that represents the classic NAFLD phenotype associated with metabolic syndrome.

## Graphical abstract

• eNOS^−/−^ mice fed a high fat diet develop NAFLD and accumulate intra‐abdominal and liver fat and display altered lipid composition.• Metformin results in decreased lipid accumulation and partly mitigates the development of NAFLD.• In vivo and ex vivo ^1^H‐MRS can quantify hepatic steatosis and response to treatment in this model.

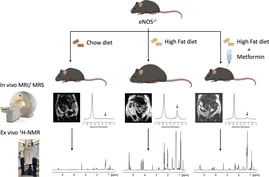

**Abbreviations used**
MAFLD: metabolic (dysfunction) associated fatty liver disease
MRI: magnetic resonance imaging
MRS: magnetic resonance spectroscopy
NAFL: nonalcoholic fatty liver
NAFLD: nonalcoholic fatty liver disease
NASH: nonalcoholic steatohepatitis
NMR: nuclear magnetic resonance
NO: nitric oxide
NOS3: endothelial nitric oxide synthase
TLC: thin layer chromatography

## 1 INTRODUCTION

Nonalcoholic fatty liver disease (NAFLD) is defined by an excessive accumulation of hepatic fat in the form of triglycerides and other lipid species.^1^, ^2^ The spectrum of NAFLD ranges from nonalcoholic fatty liver (NAFL) to nonalcoholic steatohepatitis (NASH).^3^, ^4^, ^5^ However, NAFLD can be associated with a spectrum of liver disorders, such as liver fibrosis, cirrhosis, and hepatocellular carcinoma,^6^, ^7^ and is considered the hepatic manifestation of the metabolic syndrome.^8^ NAFLD is an increasing health problem worldwide^9^, ^10^, ^11^, ^12^; however, the factors that promote the progression from NAFLD to NASH and cirrhosis remain poorly understood, although genetic variation, diet, and comorbidities such as diabetes are considered key factors.^13^, ^14^, ^15^, ^16^

Mouse models have been widely used to study NAFLD.^17^, ^18^ Special diets such as the Methionine and Choline Deficient, Choline‐Deficient L‐Amino Acid‐defined, and atherogenic diet, as well as chemical damage of the liver (e.g., streptozotocin, carbon tetrachloride, diethylnitrosamine) or genetic models (e.g., ob/ob^−/−^, ApoE^−/−^ models), combined or not, with dietary modifications, have been used to study the pathophysiology of NAFLD.^19^, ^20^, ^21^ Observations in these different models have contributed to decipher some of the mechanisms underlying the development and progression of NAFLD^21^; however, none of these approaches show the development and progression of NAFLD as observed in humans.

Nitric oxide (NO) plays an important role in the physiology and pathophysiology of the liver^22^ and its absence promotes systemic tissue alterations, including liver diseases^23^, ^24^ and steatosis. Several studies have shown that eNOS‐derived NO plays an important role in fat distribution,^25^, ^26^, ^27^ mitochondrial energy pathways, and fatty acid metabolism.^28^, ^29^, ^30^, ^31^, ^32^ Of note, hypertensive,^33^ obese, and diabetic patients, who frequently have NAFLD that evolves to NASH, show less eNOS activity and reduced NO bioavailability.^34^, ^35^ In mice, it has been shown that a deficiency of endothelial nitric oxide synthase (eNOS^−/−^, NOS3) leads to an increased accumulation of liver fat, insulin resistance,^36^ and obesity,^37^ which recapitulates several features seen in humans with metabolic syndrome and is exacerbated by Western diet‐induced hepatic inflammation and fibrosis.^25^, ^38^ In this context, eNOS^−/−^ mice represent an interesting murine model with which to evaluate features of the progression of NAFLD.

Previous studies in human and mouse liver samples using gas chromatography combined with mass spectrometry have shown that the composition of fatty acids stored in the hepatocyte changes during the progression of NAFLD.^39^, ^40^, ^41^, ^42^ Most of the studies demonstrated that a key signature of this progression is the decrease in the polyunsaturated fatty acids and increase in the monounsaturated fatty acids, while the saturated fatty acids remain almost constant.^41^, ^43^

Magnetic resonance imaging (MRI) and magnetic resonance spectroscopy (MRS) techniques are widely used for the measurement and quantification of liver fat fraction, both clinically^44^, ^45^ and preclinically.^46^ There are two general approaches, multiple point Dixon techniques^47^ and single voxel spectroscopy. For single voxel spectroscopy, STEAM is generally preferred over PRESS because shorter echo times can be used, reducing sensitivity to J‐coupling evolution; however, it remains sensitive to bias due to relaxation for different TR and TE.

The aim of this study was to assess the macroscopic body fat distribution using in vivo MRI and ^1^H‐MRS at 3 T to quantify liver fatty infiltration in eNOS^−/−^ mice fed either a high fat diet (HFD) alone or in combination with metformin (Met) treatment. Moreover, ex vivo high‐field nuclear magnetic resonance (NMR) at 9.4 T, thin layer chromatography (TLC), and histology were used for detailed analysis of mobile lipid species in untreated and treated mice.

## 2 MATERIAL AND METHODS

### 2.1 Animal studies

All procedures were performed in accordance with the guidelines of the UK Home Office.

Male wild‐type (WT) C57BL/6 mice were purchased from Charles River Laboratories (UK) and male B6.129P2‐Nos3tm1Unc/J knockout (eNOS^−/−^) mice were purchased from the Jackson Laboratory (Bar Harbor, ME, USA) and bred in our facility. WT mice were fed either a normal chow diet (ND; n = 12) or a Western diet (i.e., a HFD; n = 12) containing 21% fat from lard and 0.15% (wt/wt) cholesterol (Special Diet Services, UK). eNOS^−/−^ mice were divided into three groups: (i) the baseline group, fed a ND (n = 11) for 8 weeks; (ii) the HFD group, fed a HFD for 8 weeks (n = 12); and (iii) the HFD + Met group, fed a HFD and treated with Met administered in drinking water (dose of 50 mg/kg/day)^48^ for 8 weeks (n = 12).

All mice were individually housed. Food intake was measured during a 48‐h period once a week for 5 weeks. Food intake measurement was performed using a cage with a stainless‐steel grid without wood shavings scattered on the floor. Food was weighed prior (FW_0_) and after 48 h (FW_48_) to the nearest 0.1 g. Food intake/per day was calculated as follows:

(1) $$\text{Food intake}/\mathrm{day}=\frac{{\mathrm{FW}}_{0}-{\mathrm{FW}}_{48}}{2}$$

### 2.2 In vivo liver and abdominal MRI and ^1^H‐MRS protocol at 3 T

In vivo MRI was performed using a Philips Achieva 3‐T MR scanner (Philips Healthcare, Best, The Netherlands) equipped with a clinical gradient system (30 mTm^−1^, 200 mTm^−1^ ms^−1^). This technique was used to measure intra‐abdominal fat volume and liver ^1^H‐fat fraction. The abdomen and liver were imaged using a single‐loop surface coil (diameter = 47 mm) with the mice placed in a prone position. A cohort of animals (n = 6–8/group) were imaged at 8 weeks after commencement of the experimental protocol. Anesthesia was induced with 5% and maintained with 1%–2% isoflurane mixed with medical oxygen. For visualization of the liver, a T_2_‐weighted turbo spin echo anatomical scan was performed in the coronal plane with a field of view (FOV) = 50 x 50 x 12 mm^3^, matrix size = 112 x 112 x 12, in‐plane resolution = 0.6 x 0.6 mm^2^, slice thickness = 0.7 mm, TR/TE = 2999/80 ms, flip angle = 90°, and scan duration = 4 min 48 s. A two‐point Dixon sequence was acquired in the coronal plane to determine the intraperitoneal body fat content with a FOV = 40 x 44 x 22 mm^3^, matrix size = 224 x 224, in‐plane resolution = 0.6 x 0.6 mm, slice thickness = 1 mm, TR/TE1/TE2 = 9.4/2/3.8 ms, flip angle = 25°, and scan duration = 3 min 46 s. Fat‐only and water‐only images were obtained using the mDIXON Quant Philips package. The intra‐abdominal fat volume was calculated by manual segmentation from the fat‐only Dixon images using Osirix (OsiriX Foundation, Geneva, Switzerland) (Figure S1). Localized STEAM ^1^H‐MRS was performed in two different locations of the liver in each animal without water suppression (Figure S2) and the results were averaged. The ^1^H‐MRS acquisition parameters were: voxel volume = 5 x 5 x 5 mm^3^, TR/TE = 2/8.7 s, spectral bandwidth = 2000 Hz, spectral resolution = 1.95 Hz, NSA = 128, phase cycles = 16, flip angle = 90°, and scan duration = 4 min 52 s. Spectra were fitted with the Advanced Method for Accurate, Robust and Efficient Spectral fitting (AMARES) in the jMRUI software package.^49^, ^50^ Peak integrals were measured for the water peak at 4.7 ppm and the lipid peak at ~1.3 ppm to calculate the fat fraction as follows:

(2) $$\mathrm{fat}\_\text{fraction}=\frac{\mathrm{fat}}{\mathrm{fat}+\text{water}}$$

### 2.3 Ex vivo liver sample preparation

Following the MRI scans at 8 weeks, the mice were culled, and livers were collected for ex vivo analysis (n = 6–8/group). This technique was used to stage the progression of the liver disease. For tissue collection, the mice were anesthetized with isoflurane and perfused through the left ventricle with physiological saline, to eliminate clots and blood. The entire liver was removed and divided into smaller segments for different ex vivo analyses. For histological analysis, a portion of the liver was immediately fixed in 10% buffered formalin for 1 week at 4°C, embedded in paraffin, and sectioned (5 μm thick). For dual phase liver extraction and TLC analysis, liver samples were immediately frozen in liquid nitrogen until the analysis was performed.

### 2.4 Histology

Masson's trichrome stain (Sigma‐Aldrich, Dorset, UK) was performed in paraffin‐embedded samples to visualize liver morphology, liver fat accumulation and the presence of inflammation, ballooning, and fibrosis (n = 6). Whole slide imaging was performed using an Aperio Digital Pathology Slide Scanner (Leica Biosystems), allowing the assessment of the entire left lateral lobe, performed by a blinded pathologist. The pathologist utilized the NAFLD activity score (NAS) proposed by Kleiner et al.^51^ NASs include three main histologic features: (1) steatosis (in percentage); (2) hepatocyte ballooning degeneration (which designates a special form of liver cell degeneration associated with cell swelling and enlargement found particularly in steatohepatitis); and (3) lobular inflammation (Figure S3a). An NAS of less than 3 corresponds to an absence of NASH, while a score higher than 4 indicates the presence of NASH. An NAS of 3–4 is indeterminate.^51^

### 2.5 Dual phase liver extraction protocol for ^1^H‐NMR metabolite profiling

Tissue samples were extracted using a dual phase methanol/water/chloroform method, as previously described.^52^ Briefly, frozen tissue samples (n = 5–6) were crushed on liquid nitrogen, and ~150 mg of tissue was weighed and immediately dissolved in 2 ml each of iced methanol, chloroform, and Millipore water, then vortexed. Samples were centrifuged for 1 h at 3600 rpm at 4°C to separate aqueous, protein, and lipid fractions. The lipid layer was placed into a glass scintillation vial and left to dry at room temperature.

### 2.6 Ex vivo NMR analysis of extracted liver lipids

^1^H‐NMR spectra of lipid samples (n = 5–6) were acquired using a vertical‐bore, ultra‐shielded Bruker 9.4‐T (400 MHz) spectrometer with a broad band observe probe at 298 K. This technique was used to characterize the liver fat composition in each experimental group. Dried lipid extracts were reconstituted in 600 μl of deuterated chloroform (CDCl_3_) containing 0.05% v/v tetramethylsilane (TMS). Spectra were acquired using a pulse acquire sequence, with 64 scans, two dummy scans and 14 ppm sweep width, a repetition time of 3.5 s, 90° flip angle, 16 k datapoints and a spectral resolution of 0.34 Hz yielding an experiment duration of 3.8 min. TopSpin (version 3.5) software was used for data acquisition and for metabolite quantification. Spectra were processed with 0.5 Hz line broadening followed by automatic baseline correction. Assignment of lipid metabolites to their respective peaks was carried out based on previously obtained data, confirmed by chemical shift and with reference to published spectra.^53^ Peak areas were normalized to the TMS peaks and “apparent” lipid concentrations quantified per gram tissue wet weight (absolute quantification of individual lipids is challenging by NMR as the number of ^1^H contributing to each peak is not known).

### 2.7 Statistical analysis

Two‐group comparisons of continuous variables were performed with a Mann–Whitney nonparametric exact test after the variables were ranked. Multiple group comparisons of continuous variables were performed with a Kruskal–Wallis nonparametric ANOVA test followed by Dunn's post hoc test. Correlation analysis was performed with a Spearman test. GraphPad Prism 5.00 (San Diego, USA) was used for the statistical analysis. The data are presented as the mean ± SEM and *p* values less than 0.05 were considered statistically significant. Principal component analysis (PCA) was performed in Matlab^54^ on the NMR data from each mouse's liver fat extraction in the treated and nontreated groups, in order to identify structures in the data.

## 3 RESULTS

### 3.1 eNOS^−/−^ mice accumulate more intra‐abdominal and liver fat compared with WT mice when fed with a HFD

**FIGURE 1:**
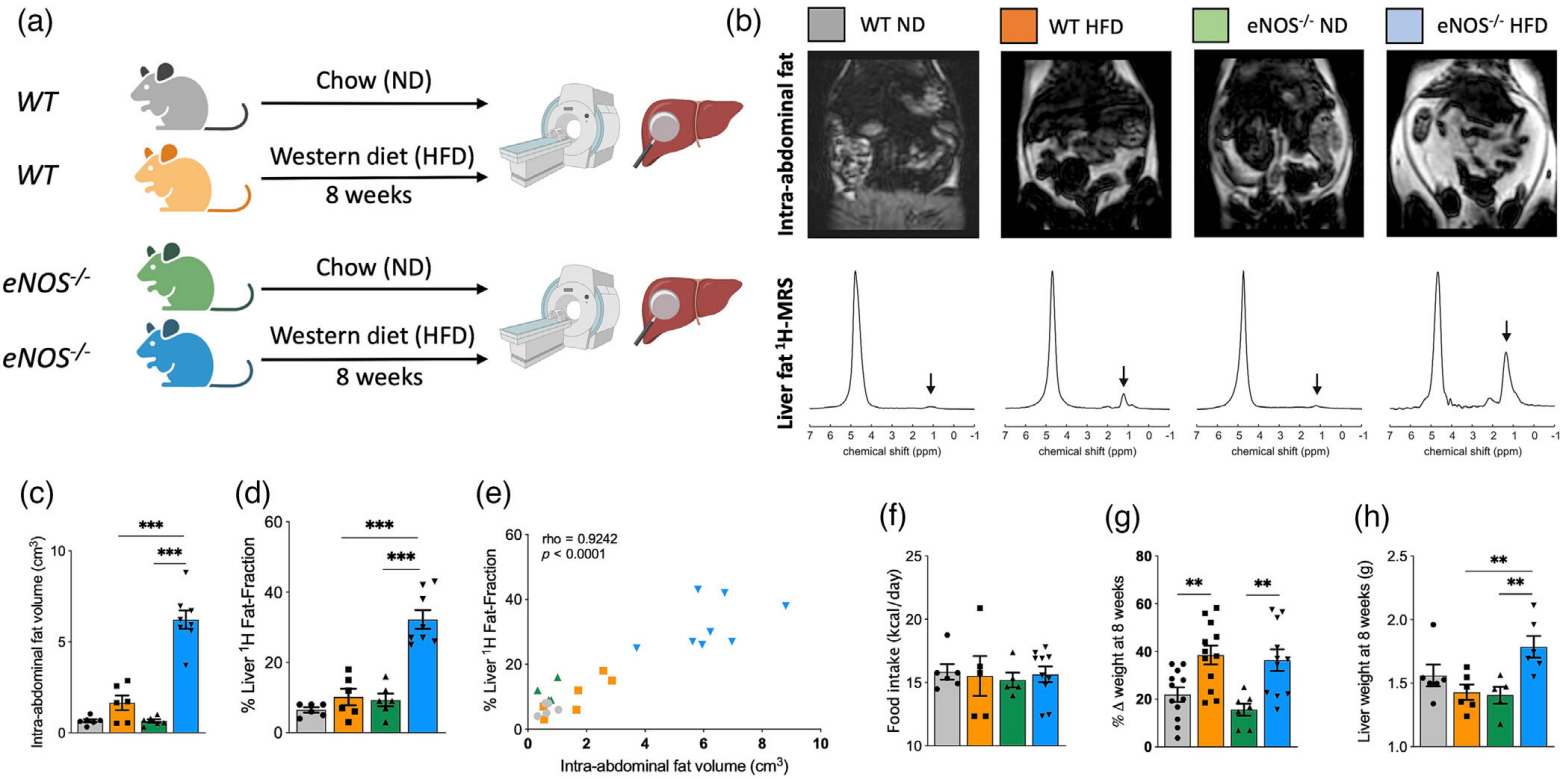
eNOS^−/−^ mice fed a high fat diet (HFD) show increased intra‐abdominal and liver fat accumulation independent of the food intake. (a) Experimental design. WT and eNOS^−/−^ mice were fed either a normal chow diet (ND) or a Western diet (i.e., a HFD) for 8 weeks, and body fat and liver fat were analyzed in vivo and ex vivo. (b) Examples of whole‐body Dixon images and in vivo voxel‐guided ^1^H‐MRS acquired at 3‐T MRI in all groups. (c) Quantification of the intra‐abdominal fat, and (d) The percentage liver fat fraction accumulated in all groups at 8 weeks (n = 6–8/group). (e) Correlation between intra‐abdominal fat volume and percentage liver fat fraction at 8 weeks (n = 6–8/group). (f) Food intake (n = 6–10/group), (g) Percentage change in body weight (n = 7–12/group), and (h) Liver weight (n = 5–6/group) in the four experimental groups at 8 weeks. Data are presented as mean ± SEM. Statistical differences are denoted by ***p < 0.001. Correlation data were analyzed with a two‐tailed nonparametric Spearman test. eNOS, endothelial nitric oxide synthase; MRS, magnetic resonance spectroscopy; WT, wild‐type.

We investigated whether the combination of a HFD and eNOS‐derived NO deficiency affected lipid and fatty acid deposition in the intraperitoneal cavity (indicator of obesity) and in the liver (indicator of NAFLD) by comparing eNOS^−/−^ mice with their WT counterparts (Figure 1a). Using in vivo Dixon MRI, eNOS^−/−^ mice fed a HFD for 8 weeks had a sixfold increase in intra‐abdominal fat volume compared with both eNOS^−/−^ and WT mice fed a ND (Figure 1b,c). Moreover, eNOS^−/−^ mice fed a HFD become obese compared with their WT counterparts, with a threefold increase in intra‐abdominal fat (Figure 1c). Liver fat fraction was calculated in all murine groups by single voxel STEAM ^1^H‐MRS using 3‐T MRI. Some spectral broadening is still evident because we did not correct for respiratory motion (or to a lesser extent cardiac motion). WT mice fed a HFD showed a trend towards increased liver fat fraction compared with WT mice fed a ND, although this was not statistically significant (Figure 1d). However, eNOS^−/−^ mice fed a HFD showed more than a threefold increase in liver fat fraction compared with eNOS^−/−^ mice fed a ND (Figure 1d). Moreover, eNOS^−/−^ mice fed a HFD showed a significant elevation of liver fat fraction compared with WT mice fed a HFD (Figure 1d), suggesting a different NAFLD progression profile in this group. Importantly, we found that the percentage liver fat fraction correlated with the intra‐abdominal fat volume at 8 weeks, showing a higher increase in both parameters in the eNOS^−/−^ mice fed a HFD (ρ = 0.92, *p* < 0.0001) (Figure 1e). This result suggests a close relation between these two biological features of the metabolic syndrome in this animal model. Importantly, food intake was also measured over the 8 weeks of the experiment, showing a similar calorie intake in all murine groups, thus discarding a potential influence of this parameter on the intra‐abdominal and liver fat accumulation measured (Figure 1f). Moreover, body weight increased in mice fed a HFD compared with those fed a ND (Figure 1g). However, the weight of the liver was increased only in eNOS^−/−^ mice fed a HFD (Figure 1h).

### 3.2 eNOS^−/−^ mice fed a HFD show an increased NAS and altered lipid liver composition

**FIGURE 2:**
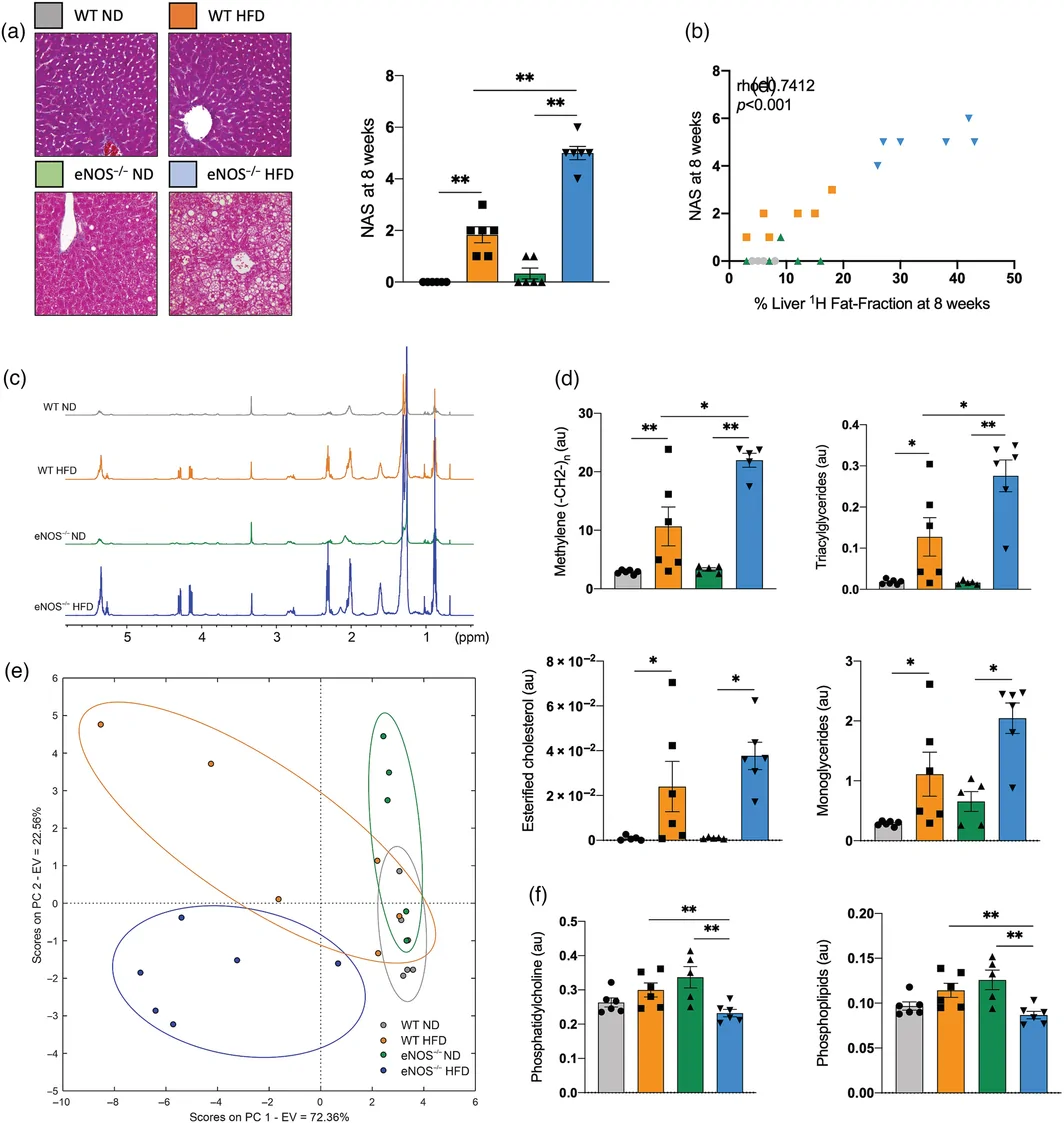
Increased NAS and altered lipid liver composition in eNOS^−/−^ mice fed a Western, high‐fat diet (HFD) compared with eNOS^−/−^ mice fed a ND and WT mice fed a ND or a HFD. (a) Representative trichrome staining and NAS quantification of the different experimental groups (n = 6/group). (b) Correlation between liver fat fraction and NAS at the 8‐week time point (n = 6/group). (c) Examples of high‐resolution ex vivo ^1^H‐NMR spectra of the liver lipids extraction from WT and eNOS^−/−^ mice fed a ND or a HFD. (d) Quantification of the methylene peak, triacylglycerides, esterified cholesterol, and monoglycerides in all groups measured by high‐resolution ^1^H‐NMR (n = 5–6/group). (e) PCA graph showing four clusters that correspond to the four groups studied (n = 5–6/group) using the data measured by high‐resolution ^1^H‐NMR. The x‐axis represents PC 1, which explains 72.36% of the total variance in the data. The y‐axis represents PC 2, which explains 22.56% of the total variance in the data. In total, the first two PCs explain almost 95% of the total variance in the data. (f) Quantification of the structural lipids phosphatidylcholine and phospholipids in all groups measured by high‐resolution ^1^H‐NMR (n = 5–6/group). Data are presented as mean ± SEM. Statistical differences are denoted by *p < 0.05, **p < 0.01. au, arbitrary units; eNOS, endothelial nitric oxide synthase; EV, explained variance; NAFLD, nonalcoholic fatty liver disease; NAS, NAFLD activity score; ND, normal diet; PC, principal component; PCA, principal component analysis; ppm, parts per million; WT, wild‐type.

To investigate whether lack of eNOS‐derived NO in combination with a HFD influences NAFLD development and progression, we next assessed the composition of the livers collected from these animals ex vivo (Figure 2). At the macromolecular level, we found that eNOS^−/−^ mice fed a HFD had increased liver weight compared with the other groups (Figure 1h). To analyze the compositional characteristics of the livers in all murine groups, we performed histology and lipid extractions that were analyzed by high‐resolution ^1^H‐NMR (Figure 2c‐f) and TLC (Figure S4). We found that livers in HFD‐fed eNOS^−/−^ mice had distinct histological features, including increased liver fat infiltration and the presence of ballooning, as quantified using the NAS compared with the other groups (Figure 2a and Figure S3a). Importantly, in vivo measurements of liver ^1^H‐Fat‐Fraction showed a significant correlation with the NAS evaluated by histology (Figure 2b). Analysis of all the peaks measured in the liver lipid profiles from high‐resolution ^1^H‐NMR spectra from the different groups is summarized in Table S1. Representative NMR spectra of the liver lipids in the four animal groups are shown in Figure 2c. The quantification of the lipid metabolites showed a significant increase in the methylene peak, triacylglycerides, esterified cholesterol, and monoglycerides in the eNOS^−/−^ and WT mice fed a HFD compared with their counterparts fed a ND (Figure 2d). Importantly, increased lipid metabolites were generally observed in mobile lipids in the eNOS^−/−^ mice compared with the WT mice when fed a HFD (Figure 2d). However, structural lipids were broadly of similar magnitude between all groups (Figure 2f). Dimensionality reduction of the high‐resolution ^1^H‐NMR using PCA into the first two principal components suggests that the four experimental conditions cluster into three groups (Figure 2e): cluster 1 with eNOS^−/−^ and WT mice fed a ND that were indistinguishable; cluster 2 with WT mice fed a HFD; and cluster 3 with eNOS^−/−^ mice fed a HFD. These results suggest that a HFD induces a change in the global lipid composition of the liver with a different impact in the eNOS^−/−^ and WT mice, which correlates with the severity of the diseases as assessed by the NAS in both groups (Figure 2a). As expected, we found that the percentage liver fat fraction correlated with the methylene peak measured by ^1^H NMR at 8 weeks, showing a higher increase in both parameters in the eNOS^−/−^ mice fed a HFD (ρ = 0.81, *p* < 0.0001) (Figure S3b). Similar results were obtained in the quantitative lipid analysis performed by TLC, showing an increase in mobile lipids in animals fed a HFD, but unaltered structural lipid composition (Figure S4).

### 3.3 Metformin treatment mitigates the accumulation of intra‐abdominal and liver fat

**FIGURE 3:**
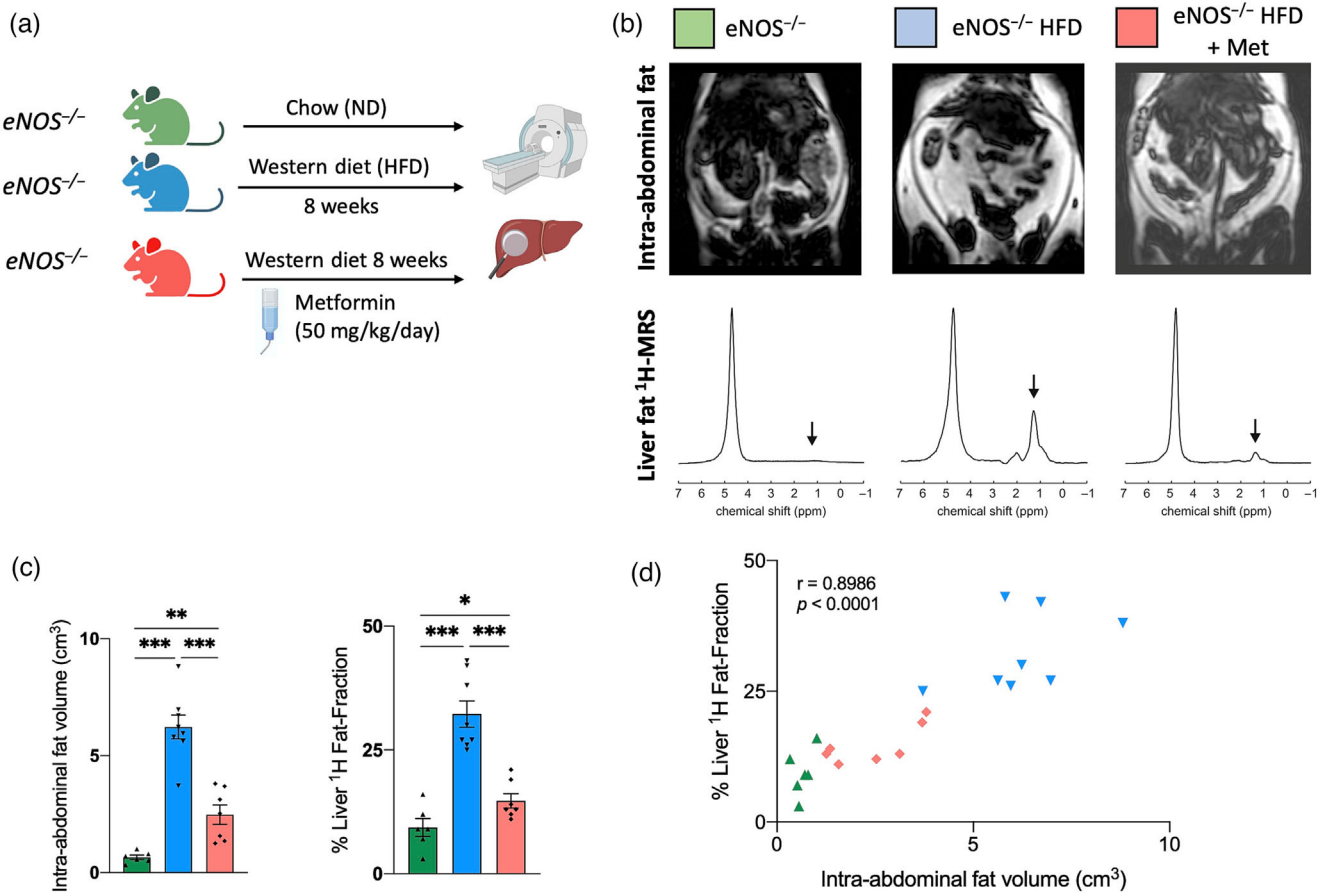
Metformin treatment decreases the intra‐abdominal and liver fat accumulation in eNOS^−/−^ mice fed a HFD. (a) Experimental design. eNOS^−/−^ mice were divided into three groups: (i) the baseline group fed a normal chow diet (ND); (ii) the Western group fed a HFD for 8 weeks; and (iii) the HFD + Met group: mice fed a HFD with simultaneous Met treatment administered in drinking water (dose of 50 mg/kg/day) for 8 weeks. Then body fat and liver fat analyzed in vivo and ex vivo was performed. (b) Examples of whole‐body Dixon images and in vivo voxel‐guided ^1^H‐MRS acquired with 3‐T MRI of treated and untreated mice. (c) Quantification of the intra‐abdominal and liver fat accumulated in all groups at 8 weeks (n = 6–8/group). (d) Correlation between intra‐abdominal fat and percentage liver fat fraction at the 8‐week time point (n = 6–8/group). Data are presented as mean ± SEM. Statistical differences are denoted by *p < 0.05; **p < 0.01; ***p < 0.001. Correlation data were analyzed with a two‐tailed nonparametric Spearman test. eNOS, endothelial nitric oxide synthase; HFD, high fat diet; Met, metformin; MRS, magnetic resonance spectroscopy.

We next investigated whether Met, a clinically used treatment for insulin resistance and type 2 diabetes, could modulate the metabolic response developed in the eNOS^−/−^ mice when fed a HFD. To this end, eNOS^−/−^ mice fed a HFD were simultaneously treated with Met (Figure 3a). In vivo MRI showed that the Met treatment significantly reduced the intra‐abdominal fat volume and the liver fat fraction compared with untreated mice (Figure 3b,c **)**. Moreover, we found that the percentage liver fat fraction strongly correlated with the intra‐abdominal fat volume at 8 weeks in both treated and untreated mice (ρ = 0.90, *p* < 0.0001) (Figure 3d).

### 3.4 Metformin shows an improved metabolic liver profile

**FIGURE 4:**
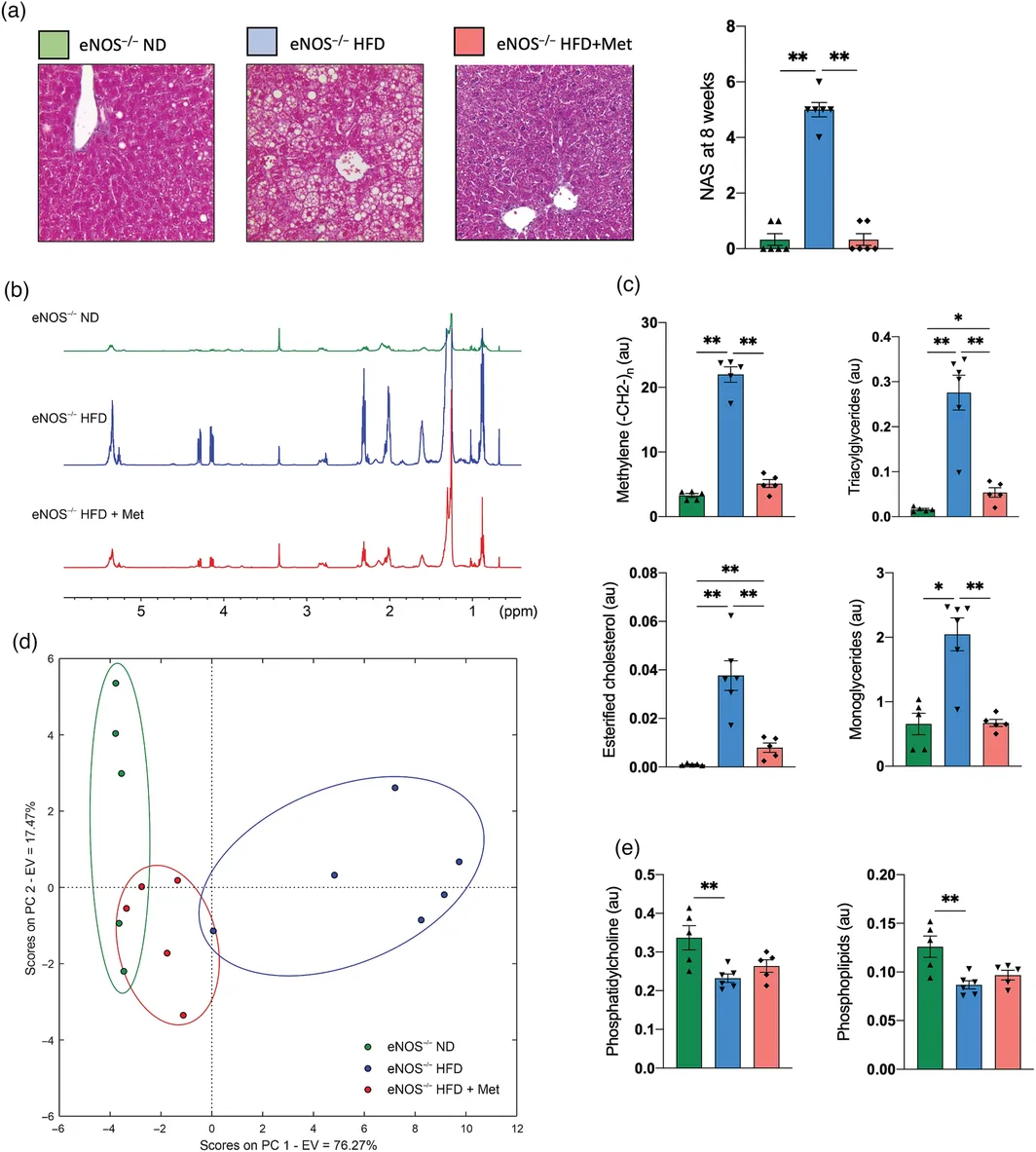
Metformin improves the liver lipid profile. (a) Representative trichrome staining and NAS quantification of the untreated and treated eNOS^−/−^ mice at 8 weeks (n = 6–8/group). (b) Representative high‐resolution ex vivo ^1^H‐NMR of untreated and treated eNOS^−/−^ mice at 8 weeks. (c) Quantification of the methylene peak, triacylglycerides, esterified cholesterol, and monoglycerides in all groups as measured by high‐resolution ^1^H‐NMR (n = 5–6/group). (d) PCA showing three clusters that correspond to the three groups studied measured by high‐resolution ^1^H‐NMR (n = 5–6/group). The x‐axis represents PC 1, which explains 72.36% of the total variance in the data. The y‐axis represents PC 2, which explains 22.56% of the total variance in the data. In total, the first two PCs explain almost 95% of the total variance in the data. (e) Quantification of the structural lipids phosphatidylcholine and phospholipids in all groups measured by high‐resolution ^1^H‐NMR (n = 5–6/group). Data are presented as mean ± SEM. Statistical differences are denoted by *p < 0.05; **p < 0.01. au, arbitrary units; eNOS, endothelial nitric oxide synthase; EV, explained variance; HFD, high fat Western diet; Met, Metformin; NAFLD, nonalcoholic fatty liver disease; NAS, NAFLD activity score; ND, normal chow diet; PC, principal component; PCA, principal component analysis; ppm, parts per million.

To explore the mechanisms by which Met treatment affects NAFLD progression and phenotype in this murine model, we quantified the histological features and liver lipid composition ex vivo. We found that Met treatment significantly decreased the NAS compared with untreated mice (Figure 4a). The analysis of the liver lipid extraction from the high‐resolution ^1^H‐NMR spectra of the treated and untreated groups is shown in Table S2. The high‐resolution ^1^H‐NMR (Figure 4b,c) showed significant differences between the eNOS^−/−^ mice fed a HFD and those receiving Met treatment. eNOS^−/−^‐treated mice showed a lowering of the methylene peak, triacylglycerides, esterified cholesterol, and monoglycerides compared with the untreated mice (Figure 4c). Structural lipids were broadly similar in magnitude in all the groups (Figure 4e). PCA showed that the eNOS^−/−^ mice fed a ND and a HFD yielded two separate clusters, and that the treated group cluster separately, showing a reversal of the liver injury produced by the HFD (Figure 4d). These results are in concordance with the histological findings and the NAS (Figure 4a). Similar tendencies were observed in the quantitative lipid analysis measured by TLC, where the Met‐treated group showed a large improvement in lipid composition, reaching values closer to that of ND‐fed mice (Figure S5).

## 4 DISCUSSION

NAFLD is one of the most important causes of chronic liver disease and it is projected to be the primary indication for liver transplantation in Western countries within the next decade.^55^, ^56^, ^57^ Important advances have been achieved in recent years in terms of diagnosis and management.^58^, ^59^, ^60^ However, noninvasive biomarkers that could predict the progression or regression of this disease are currently lacking. Because patients with NAFLD are very heterogenous, with multiple comorbidities, this has highlighted the need for advances in the analysis of each subphenotype independently. In this study we analyzed the eNOS^−/−^ model that represents a murine model of metabolic syndrome, diabetes, obesity, and NAFLD, which emulates the clinical evolution observed in a large subgroup of NAFLD patients in Western countries.^10^

We have demonstrated that: (i) eNOS^−/−^ mice fed a Western diet (i.e., a HFD) develop a progressive metabolic syndrome with an increase in intraperitoneal and subcutaneous fat accumulation that correlates with increasing liver fatty acid infiltration after being fed for 8 weeks with a HFD, showing similar progressive histological changes to those observed in humans^61^; (ii) eNOS^−/−^ mice fed a HFD present a different liver lipid composition compared with their WT counterparts, as measured by ^1^H‐NMR and TLC; (iii) ^1^H‐MRS provides metabolic information on the total liver fat content to aid the diagnosis and follow‐up of the disease progression; and (iv) the therapeutic intervention with Met reduces the body fat accumulation and decreases the liver methylene peak, triacylglycerides, esterified cholesterol, and monoglycerides and significantly improves the liver NAS.

Our experimental design enabled noninvasive and direct monitoring of disease development and treatment response in vivo. Using MRI and ^1^H‐liver MR spectroscopy, we found that eNOS^−/−^ mice fed a HFD accumulate more intra‐abdominal and liver fat than WT mice. eNOS^−/−^ livers displayed an average NAS of 5, indicating advanced liver steatosis and signs of inflammation and ballooning compared with an NAS of 2 in their WT counterparts. These results are consistent with previous studies that demonstrate that a lack of eNOS^−/−^‐derived NO impaired mitochondrial beta‐oxidation leads to accumulation of fat,^32^ which has a critical influence on lipid metabolism.^30^, ^31^, ^62^ Remarkably, many human chronic diseases, like insulin resistance, type 2 diabetes, and obesity are associated with a deficiency in the eNOS^−/−^ expression and/or function,^23^, ^63^ highlighting the role of NO in the pathophysiology of NAFLD. The current study strongly supports a causal role of NO in maintaining a healthy liver and, in its absence, promoting NAFLD progression in the context of obesity and metabolic syndrome.

We have identified a strong correlation between liver fat fraction and intra‐abdominal fat volume. Visceral adipose is a key player in the progression of liver diseases^64^ and the same correlation has previously been reported in obese children,^65^ as well as nonobese^66^, ^67^ and obese youths and adults.^68^, ^69^, ^70^ However, NAFLD also occurs in lean individuals, in whom it is characterized as hepatic steatosis with a body mass index of less than 25 kg/m^2^ (or < 23 kg/m^2^ in Asians); it has been estimated that 5% to 45% of patients with metabolic (dysfunction) associated fatty liver disease (MAFLD) are lean^71^, ^72^; therefore, there is still a need for liver biomarkers.

Detailed ex vivo characterization of the lipid composition of the liver using high‐resolution NMR and TLC showed that eNOS^−/−^ mice fed a HFD had a significantly different liver lipid ^1^H‐MRS profile compared with the HFD‐fed WT mice and ND‐fed groups. eNOS^−/−^ mice fed a HFD presented an increased concentration of mobile lipids, such as the methylene peak, triacylglycerides, esterified cholesterol, and monoglycerides. However, no significant changes were detected in structural lipids. Our findings identify a specific cluster in the PCA of the ^1^H‐NMR lipid spectra in eNOS^−/−^ mice during NAFLD progression compared with WT mice that could provide new hypotheses for the metabolic changes in the progression of this disease, and highlights the potential of ^1^H‐MRS as a noninvasive tool for diagnosis and follow‐up of patients, as other studies have suggested.^73^, ^74^ However, there is evidence that the distribution of liver damage is not homogeneous throughout the liver.^75^ However, in this study we used large voxels that practically covered the entire right or left lobe of the animal, thus it was not possible to identify differences between the different liver segments. Future studies could be developed to analyze the intersegmental distribution of liver damage. Moreover, in a clinical context, other factors such as fibrosis may be important in later stages of NAFLD, requiring a combination of more advanced relaxation mapping techniques such as T_1_, T_2_, and T_1ρ_ in addition to spectroscopy. Determining lipid and fatty acid composition will also be challenging at clinical field strengths where spectral resolution and signal‐to‐noise ratio are lower than that afforded by the high‐resolution ex vivo analysis in our study. Spectral quality (in vivo linewidths) could be improved through the implementation of motion correction techniques, either prospective triggering to synchronize the acquisition to respiratory or cardiac motion, or retrospective reconstruction using image‐derived navigators.

Metformin has many mechanisms of action. Physiologically, it acts directly or indirectly on the liver to lower glucose production. At the molecular level, Met inhibits the mitochondrial respiratory chain in the liver, leading to activation of AMPK, enhancing insulin sensitivity, and lowering cAMP, thus reducing the expression of gluconeogenic enzymes.^76^, ^77^ Metformin has a key role in hepatic glucose production and insulin sensitivity that should help in NAFLD associated with metabolic syndrome.^78^ Although Met has been shown to have a significant impact in reducing obesity and attenuating the progression of NAFLD in murine models,^79^, ^80^ their role in preventing and treating NAFLD/NASH is controversial, and therefore they do not appear in the first line of treatment.^81^, ^82^, ^83^ Recent meta‐analysis shows that antihyperglycemic drugs such as pioglitazone may have a role in the treatment of NAFLD, and the evidence for the use of Met is inconclusive.^84^ We hypothesize that most of this controversy arises from the use of different mouse models that represent different phenotypes of NAFLD, and therefore respond differently to pharmacological treatments. In our work, using a NAFLD model that is a consequence of metabolic syndrome and obesity, we showed that eNOS^−/−^ mice fed a HFD and treated with Met had a decrease in intraperitoneal fat accumulation and liver steatosis, as well as a reduction in their progression to NASH. The Met‐treated group showed an improved liver histological analysis and biomarker profile compared with the nontreated group, reaching the parameters measured in the eNOS^−/−^ mice fed a ND. Our results suggest that the quantification of the total liver fat content could be used to diagnose, stage, and assess response to treatment. However, identification and quantification of the liver lipid profile is also necessary because it could importantly contribute to expand our knowledge of the molecular mechanisms involved in disease development, progression, and treatment response. Our ex vivo studies using high‐resolution ^1^H‐NMR and TLC demonstrated that liver lipid composition provides an indirect biomarker of liver response to treatment that could be used as a surrogate biomarker of liver NAFLD/NASH regression in the context of the metabolic syndrome.

An ideal NAFLD/NASH animal model should reflect the hepatic histopathology and pathophysiology of human NAFLD/NASH. To achieve this, the use of genetically modified mice or mice fed hepatotoxic diets has been instrumental in understanding this pathology.^19^, ^85^ Although there is no perfect animal model, the eNOS^−/−^ model we used in this study exhibits many human‐like phenotypical features in the progression of fatty liver, such as metabolic syndrome with insulin resistance, obesity, and diabetes, and a histopathological progression very similar to that of humans.

In conclusion, we have demonstrated that in vivo liver MRI and ^1^H‐MRS can noninvasively stage the early stages in the development of NAFLD and monitor treatment response after administration of Met in an eNOS^−/−^ murine model of NAFLD. Our study characterizes a suitable animal model to study a subgroup of NAFLD that is associated with the metabolic syndrome, shows the potential of noninvasively diagnosing and staging NALFD progression by MRI/MRS, and broadens our understanding of the effects of existing treatments.

## CONFLICT OF INTEREST STATEMENT

The authors declare no conflicts of interest.

## Supporting information


**Figure S1.** Example of abdominal fat segmentation. **a)** Coronal T2w abdominal imaging. b) Intraperitoneal Fat only reconstruction for fat segmentation.
**Figure S2.** Coronal T2w abdominal imaging showing an example of the liver spectroscopy voxel positioning in 3 T 1H‐MRS acquisition.
**Figure S3. a)** Average NAS score per feature in each group of mice. **b)** Correlation between percentage liver fat fraction and methylene peak measure by ^1^H NMR at the 8 weeks (n = 6/group).
**Figure S4. eNOS** ^**−/−**^ **mice fed HFD shows increase accumulation of mobile lipids in the liver.** Quantification of the mobile lipids: total fatty acids, diglycerides, cholesteryl esters and triglycerides; and the structural lipids, phosphatydilethanolamine, phosphatidylinositol, phosphatidylcholine and phosphatidylserine using Thin‐layer chromatography (TLC) in all groups at 8 weeks (n = 9–10/group). Data are presented as mean±SEM. Statistical differences are denoted by, **p < 0.01, ***p < 0.001. Abbreviations: WT: wild type; eNOS, endothelial nitric oxide synthase; ND, normal chow diet; HFD, western diet; prot: protein.
**Figure S5. Treated eNOS** ^**−/−**^ **mice fed HFD shows a decrease accumulation of mobile lipids in the liver.** Quantification of the mobile lipids, total fatty acids, diglycerides, cholesteryl esters and triglycerides; and the structural lipids, phosphatydilethanolamine, phosphatidylinositol, phosphatidylcholine and phosphatidylserine using Thin‐layer chromatography (TLC) in treated and untreated eNOS^−/−^ mice at 8 weeks (n = 6–10/group). Data are presented as mean±SEM. Statistical differences are denoted by *P < 0.05, **P < 0.01, ***P < 0.001. Abbreviations: eNOS, endothelial nitric oxide synthase; ND, normal diet; HFD, high fat diet; Met, metformin; prot: protein.
**Table S1.** Lipid liver profile measured by high‐resolution ^1^H‐NMR in WT and eNOS^**−/−**^ mice fed either normal chow diet or western diet for 8 weeks. Peak integrals normalized to TMS peak per gram wet weight of tissue multiplied by number of moles TMS x 1e3.
**Table S2.** Lipid liver profile measured by high‐resolution ^1^H‐NMR in treated and untreated eNOS^**−/−**^ mice for 8 weeks. Peak integrals normalized to TMS peak per gram wet weight of tissue multiplied by number of moles TMS x 1e3.

## Data Availability

The data that support the findings of this study are available upon request to the authors.
