# Supplementary material for: Characterization of hepatic fatty acids using magnetic resonance spectroscopy for the assessment of treatment response to metformin in an eNOS−/− mouse model of metabolic nonalcoholic fatty liver disease/nonalcoholic steatohepatitis
Source: NMR Biomed. 2023 Apr 11;36(8):e4932. doi: 10.1002/nbm.4932 (PMC10704844; doi:10.1002/nbm.4932)
Supplement: Supplementary file 1 — Figure S1. Example of abdominal fat segmentation. a) Coronal T2w abdominal imaging. b) Intraperitoneal Fat only reconstruction for fat segmentation. Figure S2. Coronal T2w abdominal imaging showing an example of the liver spectroscopy voxel positioning in 3 T 1H‐MRS acquisition. Figure S3. a) Average NAS score per feature in each group of mice. b) Correlation between percentage liver fat fraction and methylene peak measure by 1H NMR at the 8 weeks (n = 6/group). Figure S4. eNOS −/− mice fed HFD shows increase accumulation of mobile lipids in the liver. Quantification of the mobile lipids: total fatty acids, diglycerides, cholesteryl esters and triglycerides; and the structural lipids, phosphatydilethanolamine, phosphatidylinositol, phosphatidylcholine and phosphatidylserine using Thin‐layer chromatography (TLC) in all groups at 8 weeks (n = 9–10/group). Data are presented as mean±SEM. Statistical differences are denoted by, **p < 0.01, ***p < 0.001. Abbreviations: WT: wild type; eNOS, endothelial nitric oxide synthase; ND, normal chow diet; HFD, western diet; prot: protein. Figure S5. Treated eNOS −/− mice fed HFD shows a decrease accumulation of mobile lipids in the liver. Quantification of the mobile lipids, total fatty acids, diglycerides, cholesteryl esters and triglycerides; and the structural lipids, phosphatydilethanolamine, phosphatidylinositol, phosphatidylcholine and phosphatidylserine using Thin‐layer chromatography (TLC) in treated and untreated eNOS−/− mice at 8 weeks (n = 6–10/group). Data are presented as mean±SEM. Statistical differences are denoted by *P < 0.05, **P < 0.01, ***P < 0.001. Abbreviations: eNOS, endothelial nitric oxide synthase; ND, normal diet; HFD, high fat diet; Met, metformin; prot: protein. Table S1. Lipid liver profile measured by high‐resolution 1H‐NMR in WT and eNOS −/− mice fed either normal chow diet or western diet for 8 weeks. Peak integrals normalized to TMS peak per gram wet weight of tissue multiplied by number of moles [file NBM-36-e4932-s001.docx]

**SUPPLEMENTARY MATERIAL**

**Thin Layer Chromatography (TLC)**

Liver samples were processed as previously described (57, 58). This technique was used to characterize the liver fat composition in each experimental group. Briefly, frozen liver samples (n=9-10/group) were homogenized using a motor-driven Potter-Elvehjem homogenizer (B. Braun Biotech International; Germany) in 10 volumes of cold PBS by 20 strokes of a Teflon pestle at 700rev/min at 4°C. After that, liver homogenate was sonicated. Then, one part of the homogenate was used for protein quantification by the BCA method, and the other part for lipid extraction. The volume corresponding to 1.5mg of liver protein was used for lipid extraction. For this, the Folch method was used (57, 58). Briefly, 1.5ml of dH_2_O (distilled water) and 8ml of chloroform:methanol:HCl (2:1:0.0075, v/v/v) mixture (Scharlau Chemicals; Spain) were added to the liver homogenates, and tubes were vigorously shaken for 2min. Lipids were separated by TLC through six chromatographic developments as detailed before (58). First, the silica-gel plates of 20 x 20cm (“Pre-coated TLC-plates SIL-G25”, Macherey-Nagel; Germany) were pre-treated with 1mM EDTA-Na_2_. Dry silica-gel plates were washed with chroloform:methanol:dH2O (60:40:10, v/v/v) in order to remove contaminants. Standards, with known lipid concentrations, were prepared from pure lipid mixtures (Avanti Polar Lipids; USA and Sigma-Aldrich; USA). When the chromatographic separations were performed, the plate was stained by immersing in a CuSO_4_ solution at 10% (w/v) in H_3_PO_4_ at 8% (v/v) for 20s. After that, the plates were dried. Then, the lipid spots were developed by heating the plates for 3min at 200°C. The image of the TLC plate was digitalized with the densitometer GS-800 and quantification was performed with the Quantity One software (Bio-Rad; USA). The integrated optic density (IOD) of each lipid spot, was interpolated in the IOD values of the calibration curves. The results obtained were expressed in nmol per mg of cellular protein.

**Supplemental Figures:**

**Supplemental Figure 1.** Example of abdominal fat segmentation. **a)** Coronal T2w abdominal imaging. b) Intraperitoneal Fat only reconstruction for fat segmentation.

**Supplemental Figure 2.** Coronal T2w abdominal imaging showing an example of the liver spectroscopy voxel positioning in 3T 1H-MRS acquisition.

**Supplemental Figure 3.** **a)** Average NAS score per feature in each group of mice. **b)** Correlation between percentage liver fat fraction and methylene peak measure by ^1^H NMR at the 8 weeks (n=6/group).

**Supplemental Figure 4. eNOS^-/-^ mice fed HFD shows increase accumulation of mobile lipids in the liver.** Quantification of the mobile lipids: total fatty acids, diglycerides, cholesteryl esters and triglycerides; and the structural lipids, phosphatydilethanolamine, phosphatidylinositol, phosphatidylcholine and phosphatidylserine using Thin-layer chromatography (TLC) in all groups at 8 weeks (n=9-10/group). Data are presented as mean±SEM. Statistical differences are denoted by, **p<0.01, ***p<0.001. Abbreviations: WT: wild type; eNOS, endothelial nitric oxide synthase; ND, normal chow diet; HFD, western diet; prot: protein.

**Supplemental Figure 5. Treated eNOS^-/-^ mice fed HFD shows a decrease accumulation of mobile lipids in the liver.** Quantification of the mobile lipids, total fatty acids, diglycerides, cholesteryl esters and triglycerides; and the structural lipids, phosphatydilethanolamine, phosphatidylinositol, phosphatidylcholine and phosphatidylserine using Thin-layer chromatography (TLC) in treated and untreated eNOS^-/-^ mice at 8 weeks (n=6-10/group). Data are presented as mean±SEM. Statistical differences are denoted by *P<0.05, **P<0.01, ***P<0.001. Abbreviations: eNOS, endothelial nitric oxide synthase; ND, normal diet; HFD, high fat diet; Met, metformin; prot: protein.

**Supplemental tables:**

**Supplemental table 1.** Lipid liver profile measured by high-resolution ^1^H-NMR in WT and eNOS**^-/-^** mice fed either normal chow diet or western diet for 8 weeks. Peak integrals normalized to TMS peak per gram wet weight of tissue multiplied by number of moles TMS x 1e3.

**Supplemental table 2.** Lipid liver profile measured by high-resolution ^1^H-NMR in treated and untreated eNOS**^-/-^** mice for 8 weeks. Peak integrals normalized to TMS peak per gram wet weight of tissue multiplied by number of moles TMS x 1e3.
